# The relationship between statistical power and predictor distribution in multilevel logistic regression: a simulation-based approach

**DOI:** 10.1186/s12874-019-0742-8

**Published:** 2019-05-09

**Authors:** Oscar L. Olvera Astivia, Anne Gadermann, Martin Guhn

**Affiliations:** Human Early Learning Partnership, The University of British Columbia, 2206 East Mall, Vancouver, British Columbia V6T 1Z4 Canada

## Abstract

**Background:**

Despite its popularity, issues concerning the estimation of power in multilevel logistic regression models are prevalent because of the complexity involved in its calculation (i.e., computer-simulation-based approaches). These issues are further compounded by the fact that the distribution of the predictors can play a role in the power to estimate these effects. To address both matters, we present a sample of cases documenting the influence that predictor distribution have on statistical power as well as a user-friendly, web-based application to conduct power analysis for multilevel logistic regression.

**Method:**

Computer simulations are implemented to estimate statistical power in multilevel logistic regression with varying numbers of clusters, varying cluster sample sizes, and non-normal and non-symmetrical distributions of the Level 1/2 predictors. Power curves were simulated to see in what ways non-normal/unbalanced distributions of a binary predictor and a continuous predictor affect the detection of population effect sizes for main effects, a cross-level interaction and the variance of the random effects.

**Results:**

Skewed continuous predictors and unbalanced binary ones require larger sample sizes at both levels than balanced binary predictors and normally-distributed continuous ones. In the most extreme case of imbalance (10% incidence) and skewness of a chi-square distribution with 1 degree of freedom, even 110 Level 2 units and 100 Level 1 units were not sufficient for all predictors to reach power of 80%, mostly hovering at around 50% with the exception of the skewed, continuous Level 2 predictor.

**Conclusions:**

Given the complex interactive influence among sample sizes, effect sizes and predictor distribution characteristics, it seems unwarranted to make generic rule-of-thumb sample size recommendations for multilevel logistic regression, aside from the fact that larger sample sizes are required when the distributions of the predictors are not symmetric or balanced. The more skewed or imbalanced the predictor is, the larger the sample size requirements. To assist researchers in planning research studies, a user-friendly web application that conducts power analysis via computer simulations in the R programming language is provided. With this web application, users can conduct simulations, tailored to their study design, to estimate statistical power for multilevel logistic regression models.

## Background

Data with dependencies due to clustering or repeated measurements are commonplace within the behavioural and health sciences [1–3]. Acknowledging these dependencies increases the complexity of research hypotheses and places new demands on the analytical methods needed to test said hypotheses [4]. From the array of statistical techniques that can handle these types of dependencies, multilevel modelling or linear mixed effects models have become commonplace, with a wide variety of applications within epidemiological, social, educational and psychological fields [5].

In spite of the popularity of these statistical approaches, the added complexity implied by them places a demand for a more sophisticated technical knowledge on the user, whether it relates to issues of estimation, interpretation or distributional assumptions of the data [6, 7]. Sample size determination falls within this spectrum of added complexity since it cannot be calculated exactly and needs to be approximated via computer simulation [8]. Maas and Hox’s [9] and Pacagnella’s [10] simulation studies provide one of the most often-cited guidelines regarding sample sizes in multilevel models where they claim that, if fixed effects are of interest, a minimum of 30 Level 1 units and 10 Level 2 units are required and, if the inferences pertain to random effects, the number of Level 2 units should increase to 50. This is sometimes referred to in the literature as the “50–30 rule” of multilevel modelling and has been used before as sample size justification for using this type of statistical method [11–13]. It is important to highlight, however, that the recommendations based on these studies pertain exclusively to issues of estimate bias. When these same sample size recommendations are used to estimate power, they generally fall short of the commonly recommended 80% [14, 15].

Not much research has been published regarding sample size recommendations for multilevel logistic regression models or other types of generalized linear models [16, 17]. Zhang and Yuan [18] looked at issues of power analysis and predictor distributions, but their recommendations are presented within the context of single-level logistic regression. Only two studies seem to directly address the issue of power within the context of mixed effects logistic regression. Moineddin, Matheson and Glazier [19] concluded that, although multilevel logistic regression shares similar characteristics to regular multilevel linear regression, there are some important differences, such as the need for much larger samples to obtain unbiased estimates when testing for cross-level interactions. They also found that, although Wald-type confidence intervals showed a more consistent 95% coverage for fixed effects, the confidence interval coverage for random effects was biased downwards resulting in an inflation of Type I error rates. Schoeneberger [20] offers a comprehensive simulation study aimed at informing researchers of issues related to sample size and power when working with multilevel logistic regression, highlighting the fact that the sample size requirements for the appropriate use of these models is much larger than what is recommended for continuous multilevel linear models [11]. In some of their studies, particularly those with medium-sized, fixed effects regression coefficients, up to 80 Level 2 clusters, each with 100 Level 1 units, were needed to yield the 80% power recommended in the literature. He also offers one of the few examples where a dummy-coded binary predictor variable is included as both a Level 1 and Level 2 predictor; showing that if a binary variable is placed in the model, it tends to require larger sample sizes than that of a continuous predictor at both levels to make sure that its power and Type I error rate fall within their nominal values of .8 and .05 respectively.

In spite of the work that has been done to document the impact that sample characteristics have on the power of multilevel logistic regression, there still remain several avenues of research. For instance, it is not uncommon to work with continuous predictors that are not normally-distributed (e.g., income) or categorical predictors with an uneven number of participants within each group (e.g., minority status). Yet most of the simulation studies published to date assume both symmetrically-distributed predictors and equal number of participants across categories [14, 21–23]. There is virtually no information regarding the power to detect either continuous by categorical or categorical by categorical interactions [24]. Commonly, power analyses are conducted by using ready-made statistical software that assumes ideal conditions (i.e., normally distributed continuous variables and balanced categorical discrete variables) for the type of the data the researchers may encounter. The nature of the predictors can, however, have a considerable impact on the power to detect an effect and the influence that the predictor distribution has on power tends to be overlooked by researchers or cannot be accommodated by current software with pre-made routines [25–27].

### The present study

In order to address these issues and following up on the recommendations for future studies suggested by Schoeneberger [20], the purpose of this article is twofold: (i) To investigate the power of multilevel logistic regression models under commonly found conditions that may violate the assumptions made in power analysis regarding the type of predictors used (e.g., non-normally distributed continuous predictors and unbalanced categorical predictors); and (ii) to provide applied researchers who may be unfamiliar with the methodology of computer simulations with a user-friendly web application so that power can be approximated at the sample and effect sizes determined by them. We hope that a point-and-click, easily-accessible web application will help promote the practice of more ‘realistic’ power analyses where the distribution of the predictors is taken into account.

With regards to the first objective, we will exemplify the influence that the distribution of the predictors has on approximated power by presenting several representative scenarios where the predictors at each Level may be skewed or unbalanced using medium and large population effect sizes. The power curves of the different types of predictors (continuous, categorical and interaction) will be compared among themselves (primarily) as well as across simulation conditions to understand the interplay between distributional assumptions, effect sizes and sample sizes.

With regards to the second objective a tutorial will be presented towards the end of the article on how to use the newly-developed web application so that simulations similar to the ones presented here can be conducted or adapted to the individual needs of each researcher.

## Method

The following two-level multilevel model was used throughout the simulations, both in its two-level equation notation and single-equation notation. Notice that this model is the same one used in Moineddin, Matheson and Glazier [19]:

$$ logit\left({\pi}_{ij}\right)={\beta}_{0j}+{\beta}_{1j}{X}_{ij} $$

$$ {\beta}_{0j}={\gamma}_{00}+{\gamma}_{01}{Z}_j+{u}_{0j} $$

1 $$ {\beta}_{1j}={\gamma}_{10}+{\gamma}_{11}{Z}_j+{u}_{1j} $$

As a single-equation model, Equation (1) can be expressed as *logit*(*π*_*ij*_) = *γ*_00_ + *γ*_10_*X*_*ij*_ + *γ*_01_*Z*_*j*_ + *γ*_11_(*Z*_*j*_*X*_*ij*_) + *u*_1*j*_*X*_*ij*_ + *u*_0*j*_ with $$ \left[\begin{array}{c}{u}_{0j}\\ {}{u}_{1j}\end{array}\right]\sim N\left(\left[\begin{array}{c}0\\ {}0\end{array}\right]\kern0.5em \left[\begin{array}{cc}{\sigma}_0^2& {\sigma}_{01}\\ {}{\sigma}_{01}& {\sigma}_1^2\end{array}\right]\right) $$ by assumption, where *i* denotes Level 1 units and *j* indexes Level 2 clusters. In (1), *β* denotes Level 1 regression coefficients, *γ* is used for Level 2 regression coefficients and *u* stands for a random effect. The coefficient *γ*_11_ refers to a cross-level interaction. This type of interaction effects is commonplace in contextual effects modelling where the level 1 predictor is an individual-level variable, such as minority status or disease exposure, and the level 2 predictor may stand for a cluster-level variable, such as neighbourhood socioeconomic status or area-level pollution [28]. For instance, consider the hypothetical scenario in which one wishes to model the odds of a person’s infection as a function of disease exposure of the patient (a Level 1 predictor) and area-level measures of pollution (a Level 2 predictor). A cross-level interaction between exposure and pollution (e.g., assuming that higher levels of pollution among those exposed to the disease raise the odds of becoming infected if the same pattern does not occur for individuals not exposed to the disease) would be an example of a contextual effects model where interaction among Level 2 predictors with Level 1 ones are needed to further understand the phenomenon being studied.

The degree of between-cluster relatedness was set through the intraclass correlation coefficient (ICC) calculated as an intercept-only model, *logit*(*π*_*ij*_) = *γ*_00_ + *u*_0*j*_, using the formula $ICC=\frac{\sigma_0^2}{\sigma_0^2+{\sigma}_e^2}$ where ${\sigma}_e^2=\frac{\pi^2}{3}$ denotes the variance of a standard logistic distribution. Medium and large effect sizes, as defined in Cohen [29], were used to populate Equation (1). The effect sizes for the binary predictor are expressed in standardized mean difference units whereas the continuous predictor ones use the correlational metric, matching the recommendations presented in Cohen [29].

- ${\sigma}_0^2=\frac{\pi^2}{7}$ (medium effect size) as the variance of the random intercept, which results in an ICC of $\frac{\frac{\pi^2}{7}}{\frac{\pi^2}{7}+\frac{\pi^2}{3}}=0.3$ and ${\sigma}_0^2=\frac{\pi^2}{7}$ (large effect size) for an ICC of 0.5
- ${\sigma}_1^2=0.3$ (medium effect size) and ${\sigma}_1^2=0.5$ (large effect size) for the variance of the random intercept.
- *γ*_10_ = 0.5 (medium effect size) and *γ*_10_ = 0.8 (large effect size) for the regression coefficient of the binary predictor .
- *γ*_01_ = 0.3 (medium effect size) and *γ*_01_ = 0.5 (large effect size) for the regression coefficient of the continuous predictor .
- *γ*_11_ = 0.3 (medium effect size) and *γ*_11_ = 0.5 (large effect size) for the cross-level interaction effect.

For the continuous predictor distribution, three levels of skewness were used: normally-distributed predictors (i.e., skewness of 0), a chi-square distribution with 5 degrees of freedom (i.e., moderate skewenss of $\frac{\sqrt{8}}{5}$) and a chi-square distribution with 1 degree of freedom (i.e., extreme skewness of $\sqrt{8}$). The levels of skewness are similar to those encountered in real datasets as reported by Micceri [30], Blanca et al. [31] and Cain, Zhang and Yuan [24].For the binary categorical predictor three conditions were studied: balanced (i.e., a 50/50 split between the incidence group marked as 1 and the no-incidence group marked as 0), a moderate imbalance (i.e., a 30/70 split with 30% of the sample showing incidence) and an extreme imbalance condition (i.e., a 10/90 split with only 10% of the sample showing incidence). Three cases were studied with some representative scenarios in an attempt to better understand the relationship between power and distributional assumptions: Case (1): A “benchmark scenario” with a standard, normally-distributed Level 2 predictor (*Z*) and an evenly-balanced, dummy-coded Level 1 predictor (*X*). A second scenario with a normally-distributed Level 1 predictor (*X*) and an extremely unbalanced Level 2 binary predictor (*Z*) and a third scenario with an extremely skewed Level 1 predictor (*X*) and a perfectly-balanced Level 2 predictor (*Z*). Medium effect sizes were used throughout. Case (2): Moderate and extremely unbalanced Level 1 predictor (*X*) with moderately and extremely skewed Level 2 predictor (*Z*). Medium and large effect sizes were used. Case (3): Moderately and extremely skewed Level 1 predictor (*X*) with moderately and extremely unbalanced Level 2 predictor (*Z*). Medium effect sizes were used. For sample sizes, the Level 1 sample sizes were set to *N*_1_ = 10, 11, 12, … , 99, 100 and Level 2 sample sizes to *N*_2_ = 10, 30, 50, 70, 90, 110.^1^ Please notice that the Level 1 sample sizes are clustered within the Level 2 sample sizes so that, for instance, in the first simulation condition there are 10 clusters, each cluster having 10, 11, 12,…,99, 100 Level 1 sample units for a total sample size of 10 clusters × 100 sample units per cluster = 1000 collected sample units. For the second condition there are 30 clusters where each of the thirty clusters has 10, 11, 12,…,99,100 units and so on for all possible combinations of Level 1 and Level 2 sample sizes.

The simulations were all conducted in the R programming language using the *simglm*, *paramtest* and *lme4* packages. Gaussian quadrature integration was used for estimation and Wald-type standard errors and *p*-values were employed to calculate the power of the fixed effects. Statistical significance for the random effects was evaluated via the recommended one-degree-of-freedom, likelihood-ratio test where a chi-square difference test is conducted between the reduced model and the extended model with the added random effects [9–11, 20]. For each combination of simulation conditions, 1000 replications were run and the proportion of statistically significant parameter estimates from the total number of simulations was calculated as the empirical power of each model. The nominal alpha of 5% was used to test the significance of the coefficients.

## Results

The results are presented in two parts. First, we present the findings (power curves) from our simulation study. Second, we describe our newly developed web application that integrates the findings from our simulation study. The R-based web application allows researchers to conduct a priori power analyses for multilevel logistic regression with binary, skewed and normally-distributed predictors.

### Fixed effects, binary level 1 predictor and continuous level 2 predictor (medium effect sizes)

**Fig. 1:**
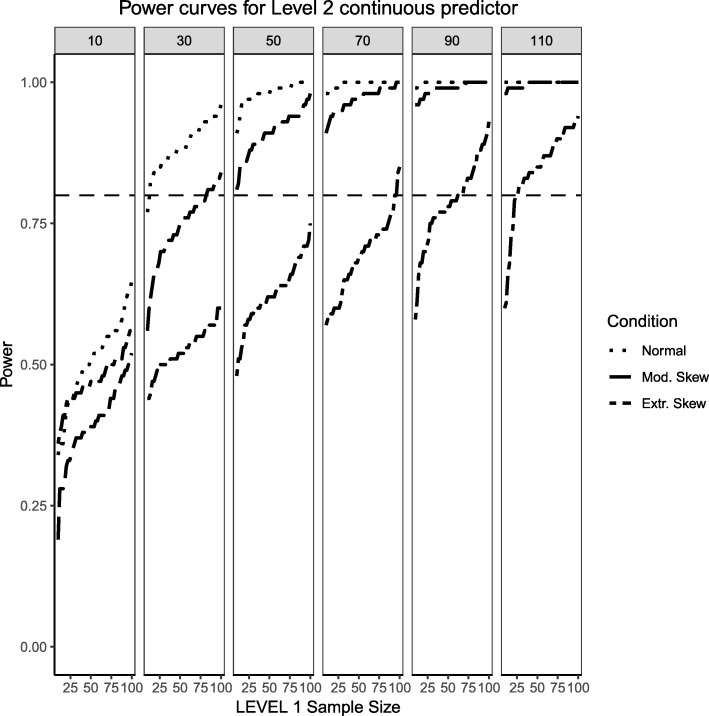
Power curves for the continuous, Level 2 predictor. Conditions of normality (‘Normal’ in the figure legend), moderate (‘Mod. skew’ in the figure legend or $\sqrt{8/5}\Big)$ and extreme (‘Extr. Skew in the figure legend or $\sqrt{8}\Big)$ skewness are presented. The population ICC is 0.3 and the regression coefficients use a medium effect size of 0.3. Power of 80% is marked with a horizontal line. The horizontal axis denotes Level 1 sample size and the vertical axis shows power. Level 2 sample sizes are shown on top of each panel (in grey). LV1 stands for Level 1 and LV2 stands for Level 2

**Fig. 2:**
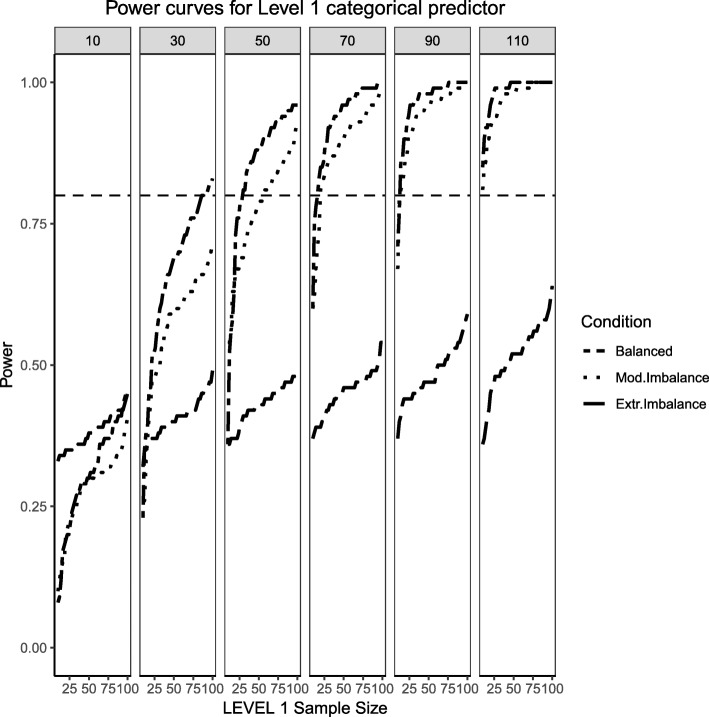
Power curves for the binary categorical, Level 1 predictor. Conditions of balance (‘Balance’ in the figure legend or 50/50), moderate (‘Mod. Imblanace’ in the figure legend or 70/30 ) and extreme (‘Extr. Imbalance in the figure legend or 90/10) imbalance are presented. The population ICC is 0.3 and the regression coefficients use a medium effect size of 0.5. Power of 80% is marked with a horizontal line. The horizontal axis denotes Level 1 sample size and the vertical axis shows power. Level 2 sample sizes are shown on top of each panel (in grey). LV1 stands for Level 1 and LV2 stands for Level 2

**Fig. 3:**
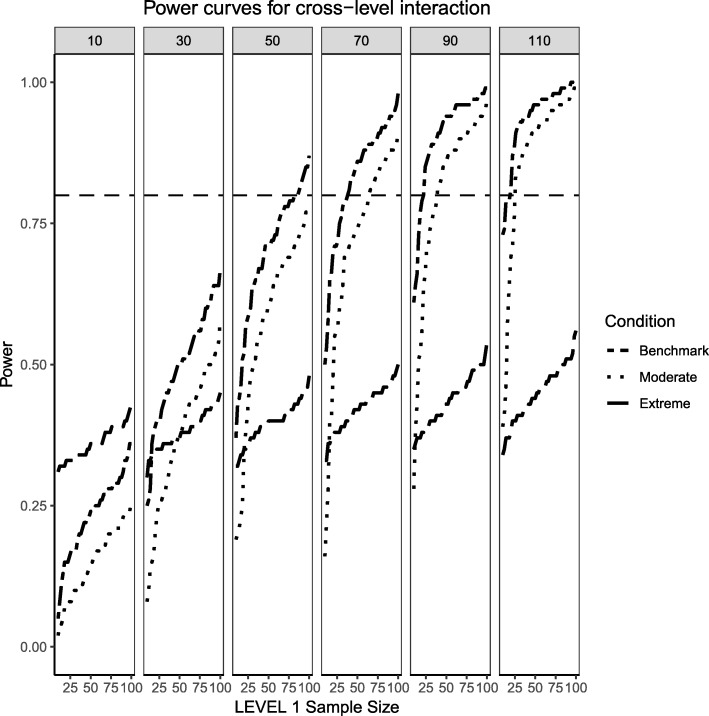
Power curves for the continuous (Level 2) by categorical (Level 1) cross-level interaction. Benchmark conditions (normally-distributed Level 2 and balanced Level 1 predictors or ‘Benchmark’ in the figure legend) as well as moderate (skewness of $\sqrt{8/5}$ and 70/30 imbalance) and extreme (skewness of $\sqrt{8}$ and 90/10 imbalance) conditions are presented. The population ICC is 0.3 and the regression coefficients use a medium effect size of 0.3. Power of 80% is marked with a horizontal line. The horizontal axis denotes Level 1 sample size and the vertical axis shows power. Level 2 sample sizes are shown on top of each panel (in grey). LV1 stands for Level 1 and LV2 stands for Level 2

Figures 1, 2 and 3 present the power curves obtained from the benchmark model (balanced Level 1 categorical predictor and normally-distributed Level 2 predictor), moderate skewness/imbalance and extreme skewness/imbalance for the continuous, categorical and cross-level interaction. The ‘benchmark model’ reflects the ‘standard assumptions’ found in previous literature [19, 20] and what one would expect most typical power analyses for multilevel logistic regression may look like. For the case of benchmark model, the power to detect an effect for the Level 2 predictor was sensitive to the number of clusters and, by the time the Level 2 sample size reached 50 or more, the detection of a medium effect approached the probability of 1. The power for the effects of the Level 1 predictor and the interaction do require interplay between Level 1 and Level 2 sample sizes. In general, larger number of Level 2 units also carry larger number of Level 1 units (given this simulation design) so that the power of Level 1 effects increases as a function of both, with the increase being more pronounced at the highest Level 2 sample size when compared to the lowest Level 2 sample size.

With the exception of the lower end of the sample size at Level 1, (around N_1_ = 30) by the time the simulation reaches 90 clusters, the power for the three types of effects was above the recommended minimum reported in Hox et al. [11].

For the moderate skewness/imbalance conditions, the degree of imbalance of the Level 1 predictor moved from 50%/50 to 70% of the sample belonging to the group coded as ‘0’ and 30% belonging to the group coded as ‘1’. For the continuous predictor, the Level 2, continuous predictor was sampled from a chi-squared distribution with 5 degrees of freedom. For the fixed effect of both types of predictors, the power was adversely affected by the increased skewness or imbalance of the predictor distribution, where larger samples both at the cluster and individual level were required in order to detect the desired effect. When the Level 2 sample size was 90 or larger, categorical and continuous main effects were detected in the vast majority of cases, but the interaction term lagged behind (N_1_ needed to be larger than approximately 30 to reach acceptable levels of power). Even at the largest Level 2 sample size of 110, the number of Level 1 units needed to be larger than 30 to ensure all three types of regression effects had a good probability of detection.

Finally, the extreme imbalance/skewness conditions (i.e., the “worst case” scenario), presents a severely skewed predictor at Level 2 (a chi-square distribution with 1 degree of freedom) and an extremely unbalanced categorical predictor at Level 1 (with 90% of the sample marked as belonging to the ‘0’ group and only 10% of the sample in the ‘1’ group). In this specific case, only the continuous Level 2 predictor has a slightly better than 50/50 chance of detecting an effect at the larger cluster sizes. Neither the Level 1 categorical predictor nor the cross-level interaction come close, only reaching power of 50% at the largest cluster size of 110 and Level 1 units close to 100.

### Random effects, binary level 1 predictor and continuous level 2 predictor (medium effect sizes)

**Fig. 4:**
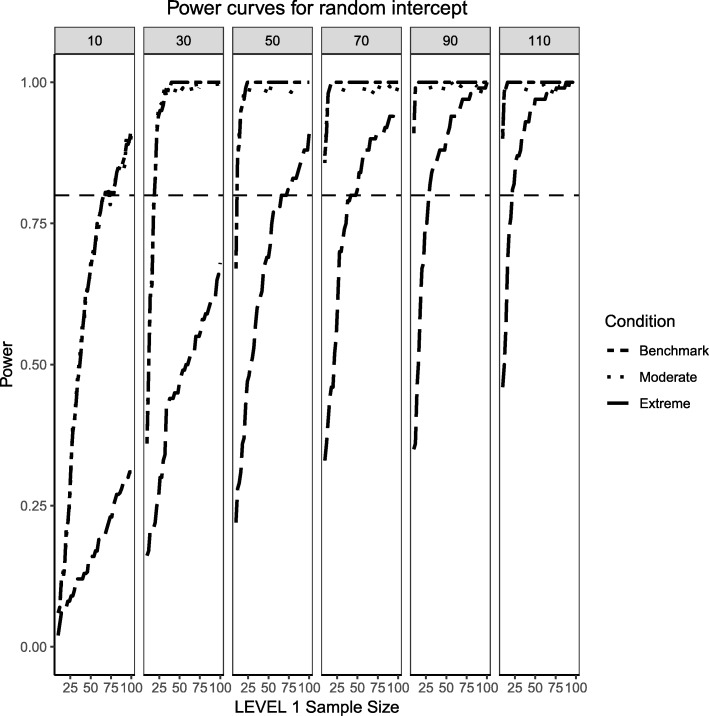
Power curves for the variance component of the random intercept are presented. Benchmark conditions (normally-distributed Level 2 and balanced Level 1 predictors or ‘Benchmark’ in the figure legend) as well as moderate (skewness of $\sqrt{8/5}$ and 70/30 imbalance) and extreme (skewness of $\sqrt{8}$ and 90/10 imbalance) conditions are shown. Variance for the random intercept is ${\sigma}_0^2=\frac{\pi^2}{7}$ for an ICC of 0.3. Power of 80% is marked with a horizontal line. Horizontal axis denotes Level 1 sample size and vertical axis shows power. Level 2 sample sizes are shown on top of each panel in grey. LV1 stands for Level 1 and LV2 stands for Level 2

**Fig. 5:**
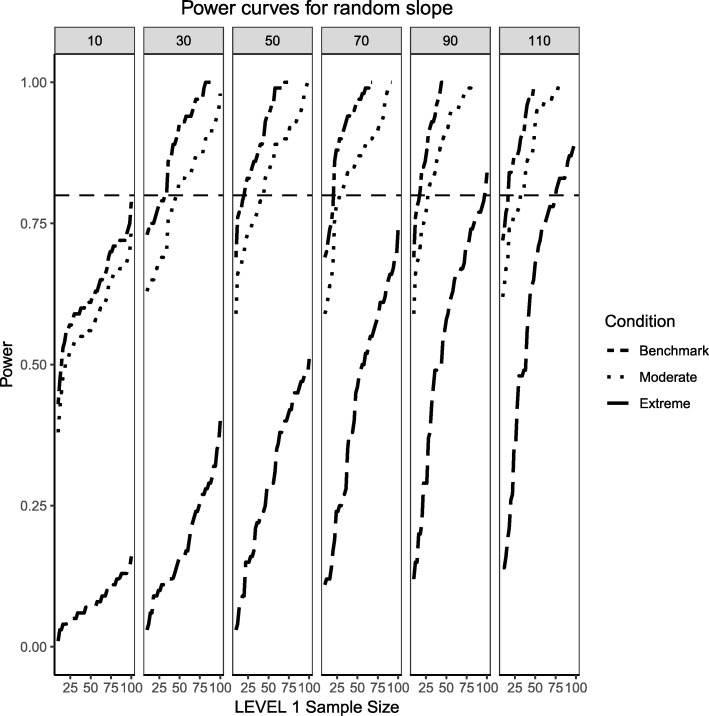
Power curves for the variance component of the random slope are presented. Benchmark conditions (normally-distributed Level 2 and balanced Level 1 predictors or ‘Benchmark’ in the figure legend) as well as moderate (skewness of $\sqrt{8/5}$ and 70/30 imbalance) and extreme (skewness of $\sqrt{8}$ and 90/10 imbalance) conditions are shown. Variance for the random slope is ${\sigma}_1^2=0.3$ with an ICC of 0.3. Power of 80% is marked with a horizontal line. Horizontal axis denotes Level 1 sample size and vertical axis shows power. Level 2 sample sizes are shown on top of each panel in grey. LV1 stands for Level 1 and LV2 stands for Level 2

Figures 4 and 5 present the power curves for the variance of the intercept and the random effects in all three simulation conditions, both evaluated via the likelihood ratio test. For the benchmark case, in both cases, the power to detect these variances depends on the interplay between Level 1 and Level 2 sample sizes. In general, the power to detect an effect for the intercept variance is higher than that of the slope variance, with the exception of lower Level 1 and Level 2 sample size conditions.

For the moderately skewed Level 2 predictor and the moderately unbalanced Level 1 binary predictor (30% of the sample labelled as “1”) the overall pattern of power curves exhibits few differences from the benchmark model condition, preserving the pattern of higher power to detect the variance of the intercept and, in comparison, lower power to detect the variance of the slope. Level 1 units also play a slightly bigger role in increasing the power to detect both effects, showing that, albeit small, the type of the distribution of the fixed effects can influence the ability to detect random effects.

Finally, the power curves for the random effects under the most severe predictor conditions of a chi-square distribution with 1 degree of freedom for the Level 2 predictor and only 10% of the sample belonging to the group labelled as ‘1’ for the categorical predictor. It appears that the increased skewness and imbalance of the predictors exert a detrimental influence on the probability of detection of the variance components, with the variance of the slope experiencing the largest shrinkage of power compared to the variance of the intercept.

### Fixed effects, binary level 1 predictor and continuous level 2 predictor (large effect sizes)

**Fig. 6:**
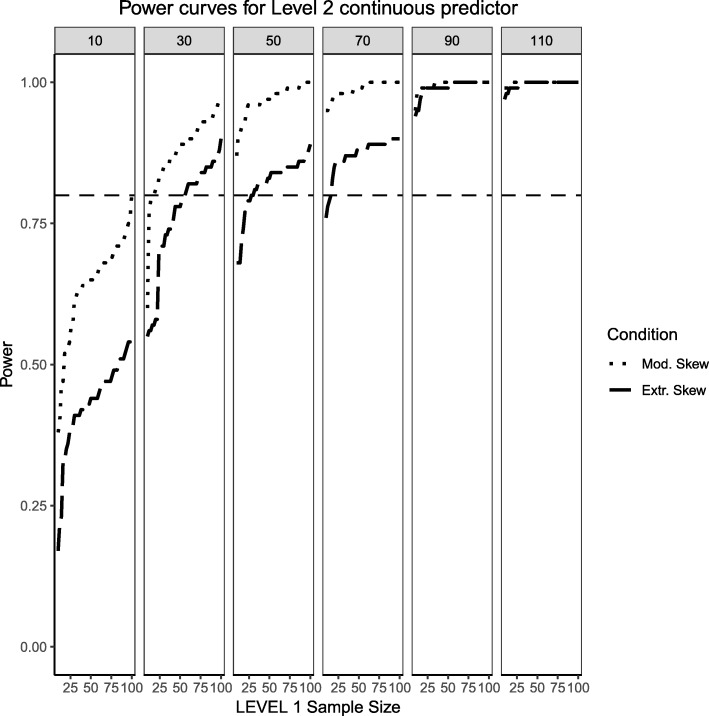
Power curves for the continuous, Level 2 predictor. Conditions of moderate (‘Mod. skew’ in the figure legend or $\sqrt{8/5}\Big)$ and extreme (‘Extr. Skew in the figure legend or $\sqrt{8}\Big)$ skewness are presented. The population ICC is 0.5 and the regression coefficients use a large effect size of 0.5. Power of 80% is marked with a horizontal line. The horizontal axis denotes Level 1 sample size and the vertical axis shows power. Level 2 sample sizes are shown on top of each panel (in grey). LV1 stands for Level 1 and LV2 stands for Level 2

**Fig. 7:**
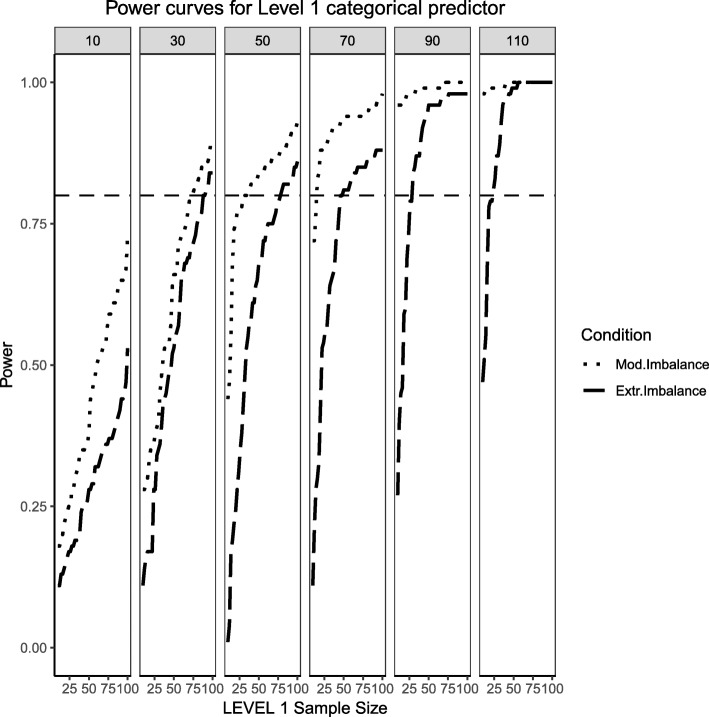
Power curves for the binary categorical, Level 1 predictor. Conditions of moderate (‘Mod. Imblanace’ in the figure legend or 70/30 ) and extreme (‘Extr. Imbalance in the figure legend or 90/10) imbalance are presented. The population ICC is 0.5 and the regression coefficients use a large effect size of 0.8. Power of 80% is marked with a horizontal line. The horizontal axis denotes Level 1 sample size and the vertical axis shows power. Level 2 sample sizes are shown on top of each panel (in grey). LV1 stands for Level 1 and LV2 stands for Level 2

**Fig. 8:**
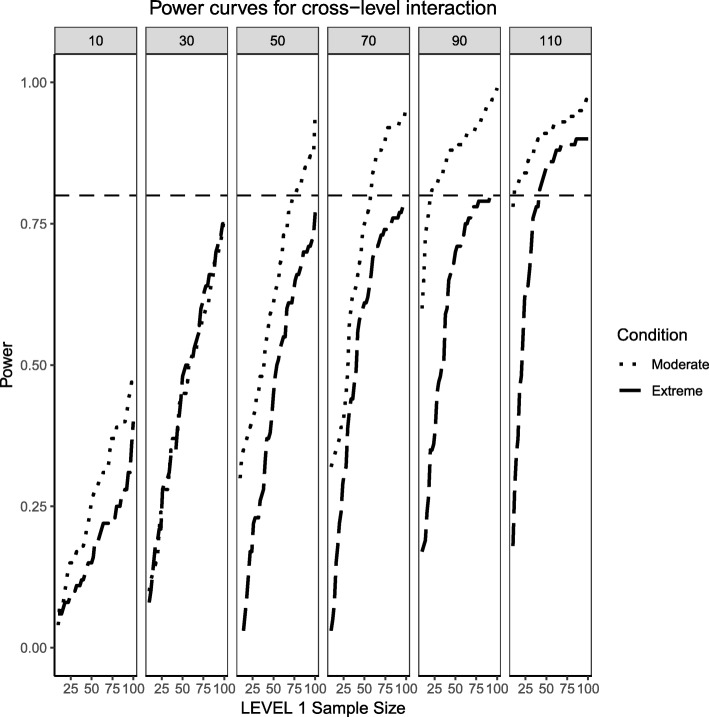
Power curves for the continuous (Level 2) by categorical (Level 1) cross-level interaction. Moderate (skewness of $\sqrt{8/5}$ and 70/30 imbalance) and extreme (skewness of $\sqrt{8}$ and 90/10 imbalance) simulation conditions are presented. The population ICC is 0.5 and the regression coefficients use a large effect size of 0.5. Power of 80% is marked with a horizontal line. The horizontal axis denotes Level 1 sample size and the vertical axis shows power. Level 2 sample sizes are shown on top of each panel (in grey). LV1 stands for Level 1 and LV2 stands for Level 2

Figures 6, 7 and 8 show the same levels of skewness of the continuous predictor and imbalance of the categorical predictor with one important exception: the population effect sizes are now large instead of medium. This helps explore how distributional characteristics interact with larger effect sizes and if they help enhance the probability of detecting an effect when compared to medium effect sizes. In general, large population effect sizes resulted in power curves with steeper slopes which also converged to the upper limit of 1 faster. They also preserve the same pattern where the continuous predictor exhibits higher probability of effect detection compared to the categorical predictor and the interaction effect. The interaction effect always showed the lowest power, although, in the large effect size condition, it does reach acceptable levels of power towards the largest Level 2 sample size conditions. The condition of extreme skewness and extreme imbalance also reveals a wider range of estimated power across Level 1 and Level 2 sample sizes. Whereas for moderate effect sizes the probability of detection was constricted and increasing slowly, for large effect sizes the slopes of the power curves were much steeper so that increases in sample sizes (at Level 1 or Level 2) were met with considerable gains in power.

### Random effects, binary level 1 predictor and continuous level 2 predictor (large effect sizes)

**Fig. 9:**
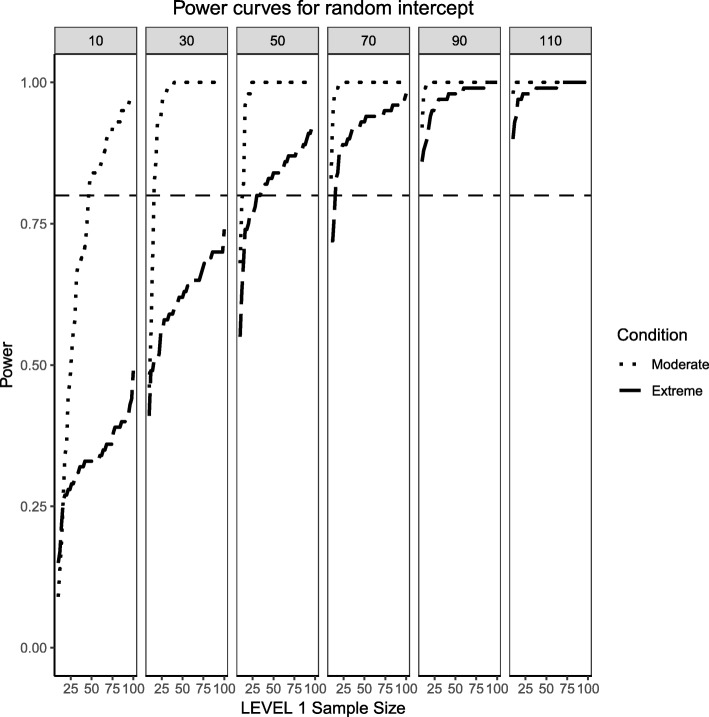
Power curves for the variance component of the random intercept are presented. Moderate (skewness of $\sqrt{8/5}$ and 70/30 imbalance) and extreme (skewness of $\sqrt{8}$ and 90/10 imbalance) simulation conditions are shown. Variance for the random intercept is ${\sigma}_0^2=\frac{\pi^2}{3}$ for an ICC of 0.5 (large effect size). Power of 80% is marked with a horizontal line. Horizontal axis denotes Level 1 sample size and vertical axis shows power. Level 2 sample sizes are shown on top of each panel in grey. LV1 stands for Level 1 and LV2 stands for Level 2

**Fig. 10:**
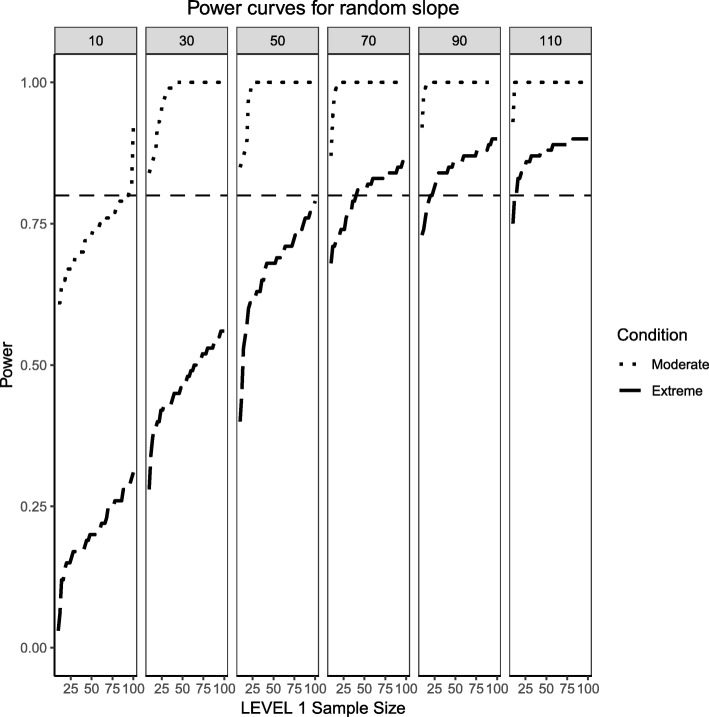
Power curves for the variance component of the random slope are presented. Moderate (skewness of $\sqrt{8/5}$ and 70/30 imbalance) and extreme (skewness of $\sqrt{8}$ and 90/10 imbalance) simulation conditions are shown. Variance for the random slope is ${\sigma}_1^2=0.5$ with an ICC of 0.5 (large effect size). Power of 80% is marked with a horizontal line. Horizontal axis denotes Level 1 sample size and vertical axis shows power. Level 2 sample sizes are shown on top of each panel in grey. LV1 stands for Level 1 and LV2 stands for Level 2

Similarly to what was shown in the previous section for the fixed effects, defining large population effect sizes for the random effects also resulted in larger probabilities of detection when compared to medium effect sizes, diminishing the negative influence that increases in skewness and imbalance had on estimated power. The overall pattern in the power curves shown in Figs. 9 and 10 remained the same for both cases, even though the power to detect the variance of the intercept was consistently greater than the power to detect the variance of the slope. It is important to point out that for the case of moderate skewness and moderate imbalance, a wider range of estimated power was observed than for the cases of extreme skewness and imbalance. This wider range, however, was only observed for the case of lower Level 2 sample sizes (i.e., N_2_ = 10, 30) and disappeared for larger Level 2 samples, where the random effects of both intercept and slope showed larger levels of power.

### Fixed effects, continuous level 1 predictor and binary level 2 predictor (medium effect sizes)

**Fig. 11:**
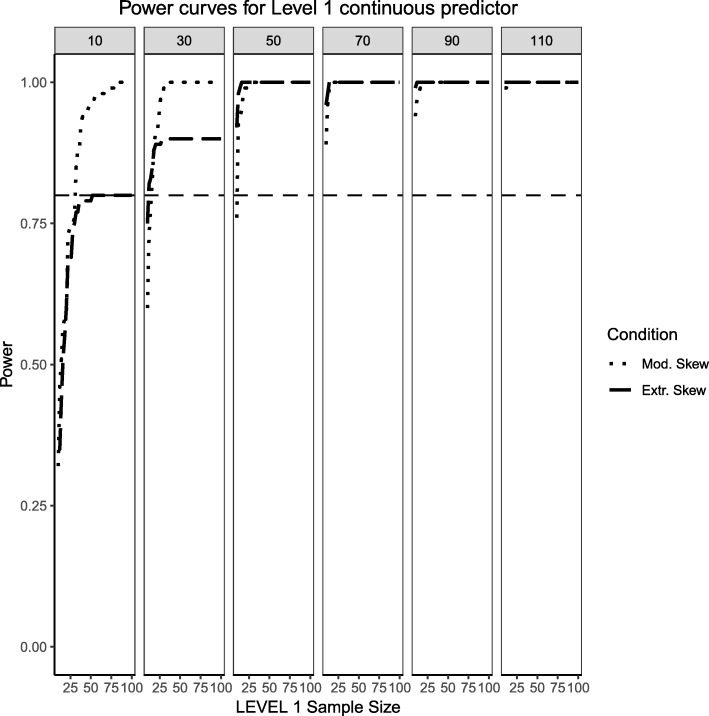
Power curves for the continuous, Level 1 predictor. Conditions of moderate (‘Mod. skew’ in the figure legend or $\sqrt{8/5}\Big)$ and extreme (‘Extr. Skew in the figure legend or $\sqrt{8}\Big)$ skewness are presented. The population ICC is 0.3 and the regression coefficients use a medium effect size of 0.3. Power of 80% is marked with a horizontal line. The horizontal axis denotes Level 1 sample size and the vertical axis shows power. Level 2 sample sizes are shown on top of each panel (in grey). LV1 stands for Level 1 and LV2 stands for Level 2

**Fig. 12:**
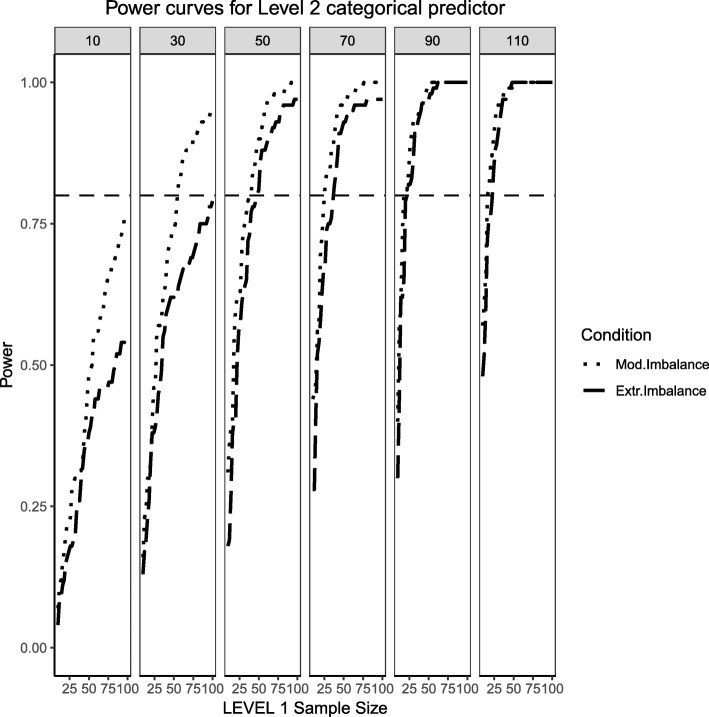
Power curves for the binary categorical, Level 2 predictor. Conditions of moderate (‘Mod. Imblanace’ in the figure legend or 70/30 ) and extreme (‘Extr. Imbalance in the figure legend or 90/10) imbalance are presented. The population ICC is 0.3 and the regression coefficients use a medium effect size of 0.5. Power of 80% is marked with a horizontal line. The horizontal axis denotes Level 1 sample size and the vertical axis shows power. Level 2 sample sizes are shown on top of each panel (in grey). LV1 stands for Level 1 and LV2 stands for Level 2

**Fig. 13:**
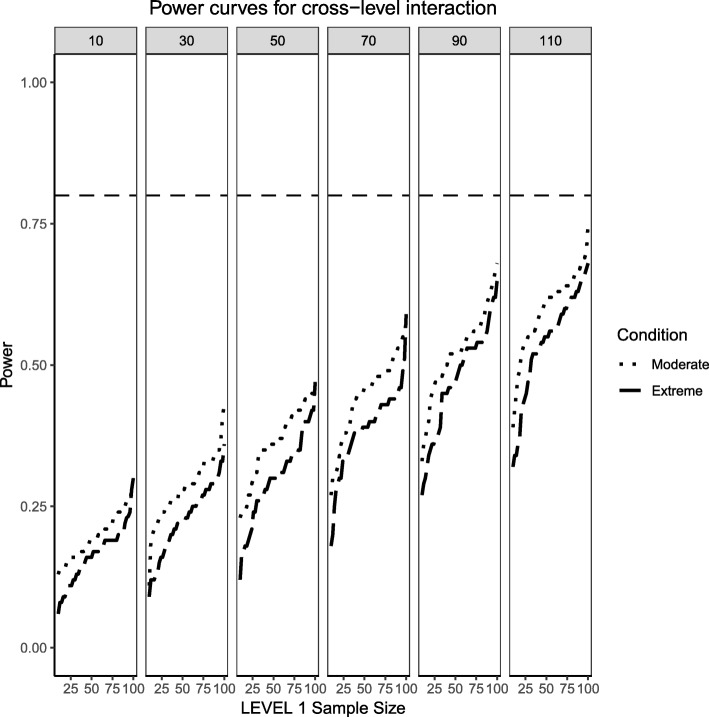
Power curves for the continuous (Level 1) by categorical (Level 2) cross-level interaction. Moderate (skewness of $\sqrt{8/5}$ and 70/30 imbalance) and extreme (skewness of $\sqrt{8}$ and 90/10 imbalance) simulation conditions are presented. The population ICC is 0.3 and the regression coefficients use a medium effect size of 0.3. Power of 80% is marked with a horizontal line. The horizontal axis denotes Level 1 sample size and the vertical axis shows power. Level 2 sample sizes are shown on top of each panel (in grey). LV1 stands for Level 1 and LV2 stands for Level 2

This section begins by showing the scenario where the Level 2 predictor is now binary categorical and the Level 1 predictor is continuously-distributed. Several important differences arose in Figs. 11, 12 and 13 when compared to Figs. 1, 2 and 3, where the distribution of the predictors switches levels. Power overall appears to be better in this present scenario, with the estimated power of both predictors and their respective interaction converging faster to their theoretical upper limit of 1 than in the scenarios presented in Figs. 1, 2 and 3. It appears that, although the continuous predictor is skewed, more sample units at the Level 1 helped it capture the relationship more efficiently. In a similar manner, although the effective sample size for the Level 2 predictor was smaller (i.e., it only depended on the number of Level 2 units as opposed to Level 1 sample size which is a product of both Level 1 times Level 2 units), with no random effect for the binary predictor, the variability of the estimates is reduced so that power to detect an effect improves. Although the power to detect the categorical by continuous interaction in both scenarios is low, it shows a moderate improvement when a continuous Level 1 predictor interacts with a binary Level 2 predictor.

### Random effects, continuous level 1 predictor and binary level 2 predictor (medium effect sizes)

**Fig. 14:**
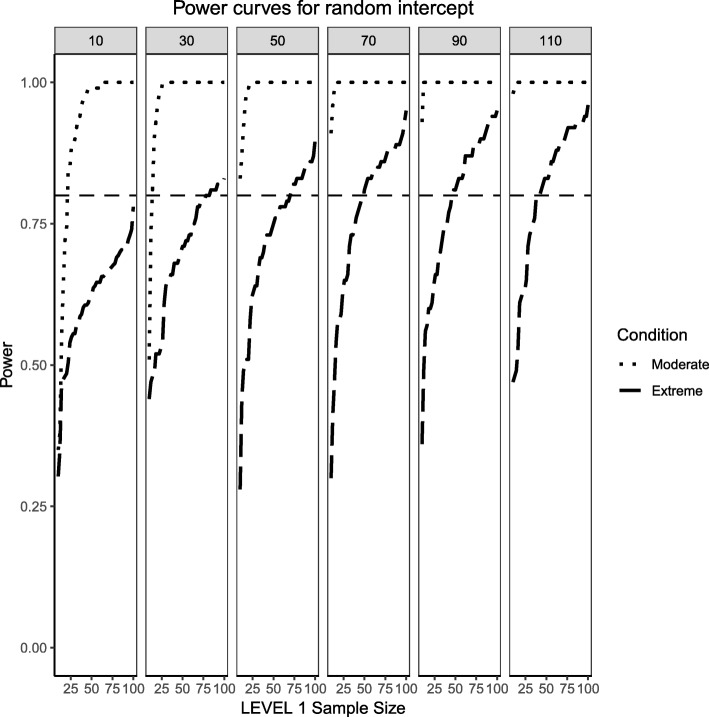
Power curves for the variance component of the random intercept are presented, now for a Level 1 (continuous) and Level 2 (categorical) predictors. Moderate (skewness of $\sqrt{8/5}$ and 70/30 imbalance) and extreme (skewness of $\sqrt{8}$ and 90/10 imbalance) simulation conditions are shown. Variance for the random intercept is ${\sigma}_0^2=\frac{\pi^2}{7}$ for an ICC of 0.3 (medium effect size). Power of 80% is marked with a horizontal line. Horizontal axis denotes Level 1 sample size and vertical axis shows power. Level 2 sample sizes are shown on top of each panel in grey. LV1 stands for Level 1 and LV2 stands for Level 2

**Fig. 15:**
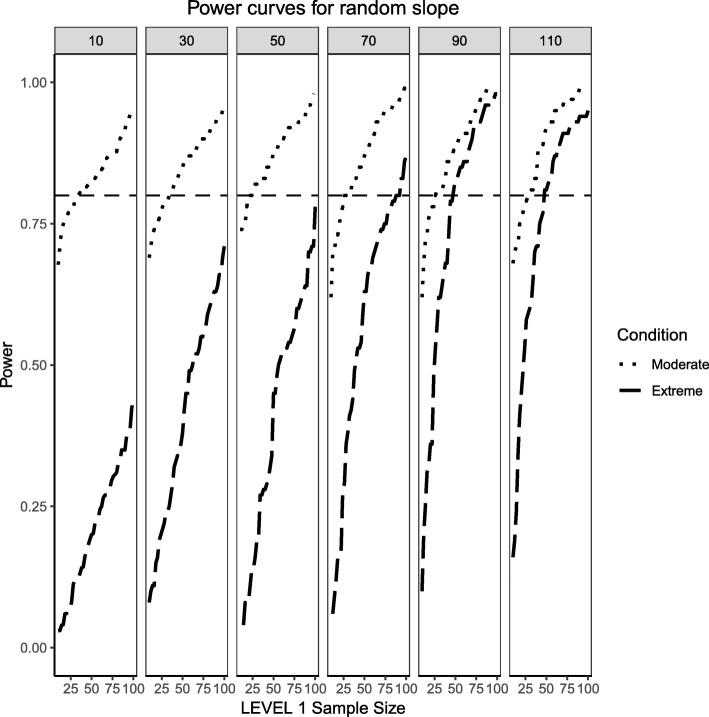
Power curves for the variance component of the random slope are presented, now for a Level 1 (continuous) and Level 2 (categorical) predictors. Moderate (skewness of $\sqrt{8/5}$ and 70/30 imbalance) and extreme (skewness of $\sqrt{8}$ and 90/10 imbalance) simulation conditions are shown. Variance for the random slope is ${\sigma}_1^2=0.3$ with an ICC of 0.3 (medium effect size). Power of 80% is marked with a horizontal line. Horizontal axis denotes Level 1 sample size and vertical axis shows power. Level 2 sample sizes are shown on top of each panel in grey. LV1 stands for Level 1 and LV2 stands for Level 2

This section also considers the condition where the Level 2 predictor is binary categorical and the Level 1 predictor is continuously-distributed to mimic the same process described above. The same levels of moderate and extreme skewness/imbalance were used. The trend of estimated power curves found for the fixed effects case is emphasized even more strongly when analyzing the random effects associated with the variance of the intercept and slope of the Level 1, continuous predictor. When comparing Figs. 4 and 5 with Figs. 14 and 15, it becomes apparent that, although the increase in skewness and imbalance still affects the probability of detection, this probability was higher for the case of random effects of continuous predictors than binary categorical predictors. This could very well be the case that the random effects are Level 1 properties, i.e., they depend on whether the low-level sample sizes change from cluster to cluster.

### Normally-distributed, level 1 predictor and extremely unbalanced level 2 predictor (medium effect sizes)

**Fig. 16:**
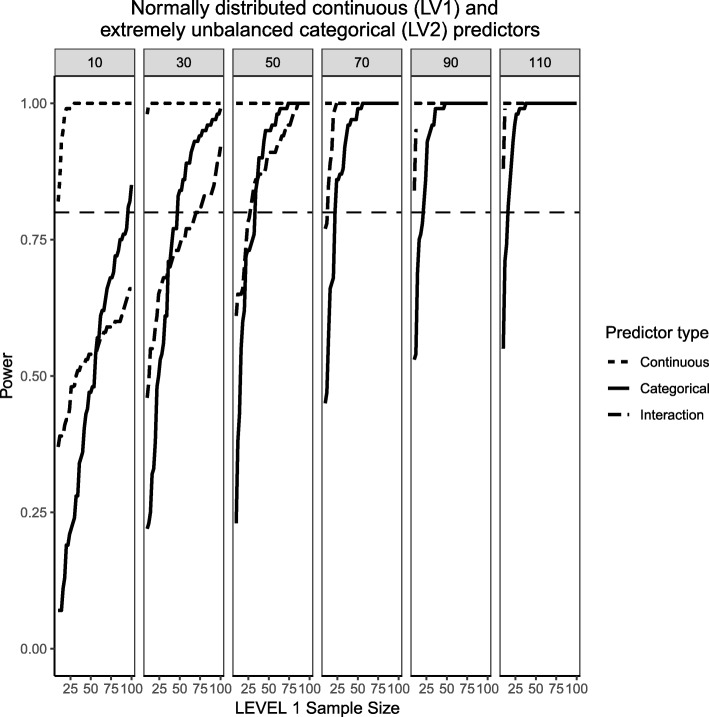
Power curves for continuous, normally-distributed Level 1 predictor and unbalanced, categorical Level 2 predictor (10% incidence). The population ICC is 0.3 and the regression coefficients use medium effect sizes (0.3 for continuous predictor and 0.5 for the categorical predictor). Power of 80% is marked with a horizontal line. Horizontal axis denotes Level 1 sample size and vertical axis shows power. Level 2 sample sizes are shown on top of each panel in grey. LV1 stands for Level 1 and LV2 stands for Level 2

In order to attempt to isolate the different influences that predictor distributions can have on estimated power and understand if the type of random variable simulated (continuous VS categorical) plays a role on estimated power, Fig. 16 present the case of a normally-distributed Level 1 predictor and an extremely unbalanced (i.e., 10% incidence) Level 2 predictor for medium effect sizes. The normally-distributed Level 1 predictor shows large estimated power across all conditions of Level 1 and Level 2 sample sizes. The usual patterns were also observed with the categorical predictor and the interaction with one important twist. As Level 2 sample sizes reached 70 clusters or more, the power to detect an interaction is higher than that of its corresponding categorical main effect. The interaction with the normally-distributed predictor (which enhances the detection of an effect) may be exerting an attenuating influence on the reduced incidence, making the power estimates higher than those of the binary predictor.

### Extremely skewed level 1 continuous predictor and balanced level 2 predictor (medium effect sizes)

**Fig. 17:**
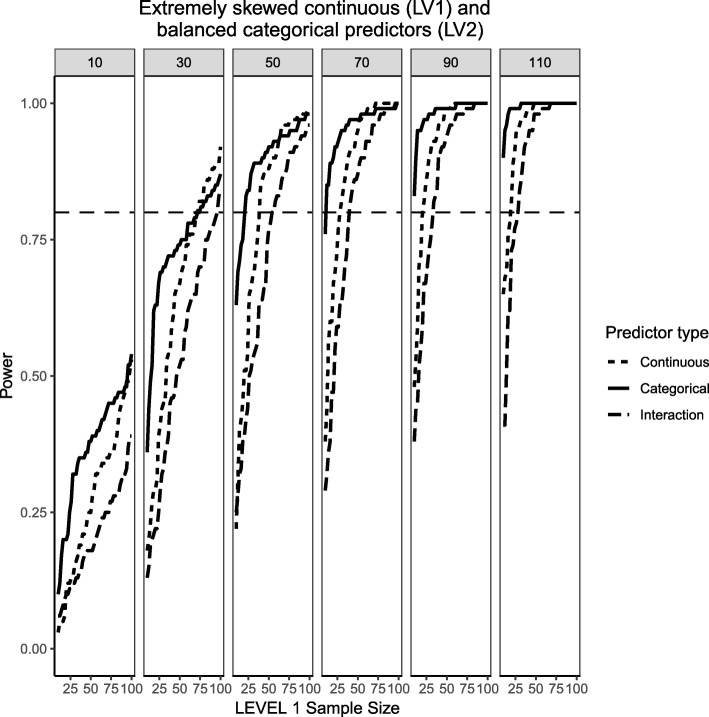
Power curves for continuous, skewed Level 1 predictor (skewness= $\sqrt{8}\Big)$ and balanced, categorical Level 2 predictor (50% incidence). The population ICC is 0.3 and the regression coefficients use medium effect sizes (0.3 for continuous predictor and 0.5 for the categorical predictor). Power of 80% is marked with a horizontal line. Horizontal axis denotes Level 1 sample size and vertical axis shows power. Level 2 sample sizes are shown on top of each panel in grey. LV1 stands for Level 1 and LV2 stands for Level 2

Finally, Fig. 17 shows the case for a Level 1, chi-square-distributed continuous predictor with 1 degree of freedom (i.e., population skewness of $\sqrt{8}$) and a balanced Level 2 categorical predictor, following on the same trend to try and understand whether the type of random variable investigated (continuous VS categorical) plays a role in power or not. This is the only case where the power of the categorical predictor is consistently greater than the continuous predictor and its interaction, although at larger Level 1 sample sizes the power of both types of predictors are very close to each other. It is also important to point out that for the skewed, continuous predictor there is a wider range of estimated power values whereas for the categorical predictor the range of power tends to shrink towards its upper bound as the number of Level 2 units increases. For instance, after 50 Level 2 units the power is consistently over 50% and at the largest Level 2 sample size of 110 it is almost always 1. The interaction still shows lower power than its corresponding main effects, albeit it gets closer and closer in power as the sample sizes at Levels 1 and 2 increase. It appears that having a skewed Level 1 predictor combined with a balanced categorical predictor results in lower power estimates than a normally-distributed Level 1 predictor with an extremely unbalanced Level 2 predictor. This echoes the previous explanations of the difficulties associated with categorical predictors where the ability to detect an effect is negatively impacted by the number of sample units that exhibit the effect.

#### Implications of results: a new web application to conduct power analysis for multilevel logistic regression

Currently, researchers must conduct computer simulations to estimate power for multilevel logistic regression models. This creates a technical barrier between applied researchers and best practices in data analysis. Yet, researchers must implement best practices when planning research studies (including sample size determination) and when applying for research grants. The current lack of availability of a user-friendly computer simulation software that can estimate power for predictors with (commonly encountered) non-normal/unbalanced distribution characteristics and cross-level interactions may push researchers to rely on questionable rules of thumb to justify/plan their sample sizes.

In order to address this issue, we have created a freely available web application developed in the R package *shiny* that provides a user-friendly, point-and-click interface to run the simulations in the present article. Researchers can try their own combinations of Level 1 and Level 2 sample sizes, effect sizes for regression coefficients and variance components in order to calculate statistical power for the specified fixed and random effects. In line with the approach in the present simulation study, users can also change the distribution of the predictors under study. For continuous predictors there are two options: normally-distributed or skewed (i.e., chi-square distributed with 1 degree of freedom). For the binary categorical predictor, the user has the option of selecting the proportion (from 0 to 1) of Level 1 units coded as ‘1’. If researchers are well-versed in the R programming language, the source code running the simulations is also provided so that it can be downloaded and modified at will.

The computations involved in approximating the power for these types of models can place an undue burden on the server where the web application is hosted. This is especially true if a large number of people are accessing it simultaneously. An alternative solution would be to run small number of replications (perhaps 100 or 500) multiple times and take the average of these power estimates. Running 10 simulations with 100 replications and taking the average of those 10 simulations is equivalent to running 1 simulation with 1000 replications. The benefit of taking the small-number-of-replications approach is that the demands placed on the server are lower, preventing potential crashes. In general, one requires a large number of replications to ensure the reliability of simulation findings. Although the default is set at 10 replications, this is merely as an example and not sufficient for research purposes.

The *shiny web application* with instructions and full tutorial can be found in:

https://psychometroscar.com/2018/07/31/power-analysis-for-multilevel-logistic-regression/

Although not currently available within the web application, the personal github account of the first author hosts R code capable of running uneven Level 1 sample sizes within Level 2 clusters to extend the applicability of this simulation-based approach to power analysis. A link to the R code is provided in the same webpage where the tutorial is hosted. It is also currently not possible for the web application to approximate a full power curve, given the unreasonable amount of time that it would take the server to do this, but the R code provided can offer this to the user if it were to be run in a local computer.

## Discussion

The popularity of multilevel or linear mixed effects models to analyze clustered data and investigate complex hypotheses has placed an increased demand on the technical knowledge of researchers interested in using them. Because no closed-form formulas are available, power analyses for multilevel logistic regressions can only be approximated through computer simulation [11, 32]. Even though we used a (relatively) simple and common model that reflects realistic data analysis conditions, the simulation results highlight the substantial influence that predictor distributions may have on the statistical power for main effects and interaction effects. In the following, we discuss several trends from our results that we believe are important to highlight.

First, categorical predictors require larger sample sizes to reach acceptable levels of power than continuous predictors. This is not a new finding within the multilevel model literature for both linear and logistic regression but, to the authors’ knowledge, this is the first simulation attempt that manipulates the proportion of prevalence in the Level 1 predictor [14]. The presence of unevenly-distributed groups is commonplace in epidemiological-observational studies, and the simulation results presented herein highlight the fact that unbalanced Level 1 predictors can substantially reduce the statistical power for the detection of an effect. Power analyses that are conducted without taking this aspect into consideration may result in over-optimistic power estimates. Although the distribution of the continuous predictor influenced the approximated power, it was mostly negligible with the Level 2 coefficient showing acceptable levels at 50 clusters or more.

Second, cross-level interactions require larger samples at both levels and the sample size demands are usually higher than those of its constituent main effects. This has also been demonstrated previously in the literature for multilevel models, but this simulation attempts to highlight the cases where the ability to detect interactions is further influenced by the distributions of the predictors that define it [20]. This information is particularly relevant to researchers who examine hypotheses within models that involve theoretically and practically relevant cross-level interactions [18].The results presented here highlight the fact that large sample sizes may be needed to detect a given effect size in this kind of setting.

Third, we recommend researchers to conceptualize their power analyses in terms of curves as opposed to single point estimates. The computational time needed to obtain a power curve might be cumbersome, due to the fact that a full simulation is needed for each combination of conditions; however, power curves allow researchers to see how the power of each predictor behaves in combination with other predictors in the model. Such information would be invaluable for planning Level 1 and Level 2 sample sizes and for making adequate inferences about levels and effect sizes for each predictor. Because two different types of sample sizes play a role in these analyses (the individual-level and cluster-level one), there is more than one combination of them that, for a given population effect size, would yield the same power. Whenever possible, using power curves for meaningful combinations of sample and effect sizes is recommended.

Finally, in light of our findings we recommend that researchers who conduct studies requiring multilevel logistic regressions with unbalanced/non-normal predictors to very judiciously choose a study’s hypothesized effect size estimates, based on existing research evidence – ideally, by drawing from comprehensive literature reviews or consulting published meta-analyses – before a power analysis is conducted [33]. This is important, because researchers commonly default to Cohen’s generic effect size categorization [29] and a hypothesized effect size that is too small or too large – however, defaulting to a general benchmark may provide an overly conservative or overly liberal estimate of power, particularly if small or medium effect sizes are paired with unbalanced groups or skewed predictors. Ideally, an informative combination of power curves at different population effect sizes should be calculated so that researchers can observe the power-sample size trade-off more directly and make practical decisions accordingly.

## Conclusion

Power analysis in multilevel modelling requires a nuanced understanding of how the statistical models are defined and estimated. Thanks to the advances of modern computers, it is now possible to calculate these power analyses, but more research is needed both in the type of predictors and how they interact in situations with non-normal and/or unbalanced distributions. For instance, creating an imbalance in dummy-coded categorical predictors induces a correlation between this predictor and its Level 2 counterpart, a simulation factor that we did not investigate but which is known to influence the power to detect an effect [34]. In our simulation, we exclusively worked with a binary predictor, and it would be of interest to future users to see how this generalizes to predictors that are coded for multiple groups. We highlighted that the level of imbalance of the categorical predictor or skewness of the continuous predictor were related to a decrease in approximated power, but only a descriptive relationship is of this fact is offered in the present article. Elucidating this point from a more mathematically-justified perspective would help users understand the relationship between distributional assumptions and power in a more proper fashion. Also, it remains to be seen in what ways introducing additional (and possibly correlated) Level 1 and Level 2 predictors affect the statistical power of Level 1 and Level 2 predictors with non-normal/unbalanced distributional properties, in the multilevel logistic regression case. Although the definitions of “small”, “medium” and “large” effect size are commonplace within the scientific literature, the simulation design presented herein does not account for the changes in the variance of the predictors (e.g., a variance of .25 for the 50/50 binary condition VS .09 for the 90/10 unbalanced condition or a variance of 1 for the standard normal case VS a variance of 2 for the chi-square distribution with 1 degree of freedom). An important avenue of future research could include a design that controls for this fact as well, given the relationship between issues of variability and power, particularly at smaller samples. Finally, multilevel logistic regression is becoming relatively well-known among researchers, but there are other multilevel generalized linear models (such as Poisson regression or Negative Binomial regression) which have received far less attention with regards to the power to detect their effects and the influence that predictor distributions have on it. This could be an interesting avenue of future research to help complement the literature of multilevel models and their sample size requirements. We hope that the findings from our simulation and the newly developed interactive power web application supports researchers in obtaining estimates of power in multilevel logistic regression without resorting to “one-size-fits-all” solutions, and also informs further theoretical and applied research in this complex and growing area of research.

## Abbreviations

LV: Level
N: Sample size
N_1_: Sample size for Level 1 units
N_2_: Sample size for Level 2 units

## Footnotes

1 Preliminary simulations were conducted to ensure that Type I error rate was maintained. Symmetric distributions with non-zero kurtosis were also examined. No detrimental effect on the power of the tests was found from these distributions.
